# The HPV Cellular Transactivator Brn-3a Can Be Used to Predict Cervical Adenocarcinoma and Squamous Carcinoma Precancer Lesions in the Developed and Developing Worlds

**DOI:** 10.1155/2009/359457

**Published:** 2010-03-09

**Authors:** Daniel Ndisang, Felipe Lorenzato, Michael Sindos, Ashfaq Khan, Albert Singer, David S. Latchman

**Affiliations:** ^1^Medical Molecular Biology Unit, Institute of Child Health, University College London, 30 Guilford Street, London WC1N 1EH, UK; ^2^Instituto Materno Infantil de Pernambuco (IMIP), Rua dos coelhos, 300 Boa Vista Recife 1393, Pernambuco, Brazil; ^3^Department of Women's and Children's Health, The Whittington Hospital, Highgate Hill, London N19 5NF, UK; ^4^Birkbeck, University of London, Malet Street, London WC1E 7HX, UK

## Abstract

The cellular transactivator Brn-3a has previously been shown to be expressed at elevated levels in the cervix of women with squamous cell carcinoma of the cervix (SCC) and to activate the expression of HPV E6 mRNA. In this study, we show that common and rare cervical precancer lesions, including those of adenocarcinoma (AC), which are usually difficult to diagnose using classical procedures, also expressed high levels of Brn-3a and can be diagnosed by measuring the levels of Brn-3a and E6 mRNAs.

## 1. Introduction

Cervical cancer is the second most common gynaecological cancer worldwide, with developing countries having the highest incidence [1]. Of the approximately 0.5 million new cases reported worldwide each year, over a quarter of a million are from Asia, with Latin America and Africa accounting for more than an eighth of a million cases [2–4]. In developed countries, cervical cancer screening programmes have helped to reduce the incidence of some of the common cervical ailments such as squamous cell carcinoma (SCC) [1–4], but not of cervical adenocarcinoma (AC) which is rapidly on the increase [5–7], or the rare sarcomatoid squamous cell carcinoma (SSCC) whose prognosis is very poor [8–11]. 

Unfortunately, in developing countries such as Brazil, cervical cancer screening programmes are too costly. As a result, unsuitable less costly cervical programmes are often put into place, thus mortality rates due to AC and other associated cervical ailments have been on the increase [12–14], and this is a common universal public health problem across the developing world [9, 10]. 

It is therefore imperative for appropriate and cost-effective diagnostic programmes which could detect the early onset of AC, SCC, and SSCC to be made available in both the developed and developing worlds [5–7, 15–17], thereby reducing the huge cost of cervical treatment and palliative programmes which is adversely affecting the public health policies of these countries [3, 15, 16, 18]. 

Interestingly, several studies have portrayed HPV as a pivotal aetiological agent in AC [5–10, 19, 20], and WHO has also indicated HPV as the primary cause of SCC and associated cervical ailments [1, 5–10]. Moreover, some of these studies have shown that, AC, SCC, and even SSCC shared a similar aetiological profile that depends on the expression of the oncogenic early-open-reading frame (EORF) E6/E7 products of HPV [3, 5–7, 9, 10]. 

Although studies have suggested that HPV could be the main aetiological determinant for AC and SCC [5–7], others have suggested environmental factors and/or genetic predisposition [8]. Nevertheless, over 90% of AC could be associated to HPV [3–6], and a very small percentage, could potentially be attributed to environmental cofactors such as smoking or medication such as oral contraceptives, or even the impact of other sexual transmitted diseases (STD) [6, 7]. 

Many studies have shown that HPV depends on its EORF E6/E7 oncoproteins [21–24] to transform cervical cells. Interestingly, cellular factors bind specific motifs of the viral URR (upstream regulatory region) to regulate the production of these oncoproteins. Brn-3a is a member of the POU family of transcription factors, expressed in cervical cells, and whose presence is closely associated with HPV mRNA positivity. [25–29]. We have previously shown that measurement of Brn-3a mRNA levels in cervical smears using our simple noninvasive cost-effective procedure has considerable potential in screening and diagnosis of cervical neoplasia in both developing and developed countries [18, 30–32]. 

In addition, several of our studies have shown not only that Brn-3a mRNA is elevated uniformly in the cervix of women with cervical ailments [33], but also that this elevation directly correlates with HPV activity and disease progression in the affected region where HPV is present [18, 27, 33]. This is because Brn-3a transactivates the HPV URR and the transcribed EORF E6 product is crucial for cervical transformation [27–29]. For instance, cervical carcinoma precursor lesions such as those of SCC and AC, which are frequently situated in the innermost regions of the cervix [6, 7], where it is always difficult to exfoliate cells for diagnosis using classical tools [6, 7, 34], could potentially be diagnosed using the mRNA levels of Brn-3a as well as HPV. This is because the oncogenic gene products of HPV, E6, and E7 depend on the activity of specific cellular transactivators such as Brn-3a for its transactivation [6, 18, 30–32]. 

Importantly, the level of E6 mRNA, which parallels that of Brn-3a in cervical scrapes, has previously been shown to have considerable potential in screening and diagnosis of cervical abnormalities [18, 30–32]. Moreover, because of the cost-effectiveness of measuring Brn-3a and E6, it could therefore be of particular importance in countries where cost is hampering cervical programmes [13, 16, 18, 34] or where classical procedures are inadequate in diagnosing the early stages of these diseases [6, 12, 31, 34]. 

Here we report that the cellular transcription factor Brn-3a measured from cervical scrapes of women with AC and SCC precancer lesions can potentially be used for the screening and diagnosing of AC and SCC. This is the first time in which the activity of the primary aetiological agent of two different types of cervical cancer has been evaluated on the basis that the cellular factor, which regulates production of its oncogenic transforming proteins [27, 35], is a logical and cost-effective target that could be used for the diagnosing, controlling, and managing of these cancers [18, 32]. 

## 2. Material and Methods

### 2.1. Patients

The present study comprised two different groups of patients. Group one is made up of patients in Recife-Brazil a developing country, and Group two comprises of patients in London-England in the United Kingdom, a developed country. The study group in Recife-Brazil, consisted of 85 women with cervical abnormalities attending one of the following gynaecological units: (1) Instituto Materno Infantil De Pernambuco (IMIP) Recife-Pernambuco Brazil, (2) The Gynaecological Oncology Research Unit Bar de Lucena Hospital-Recife, and (3) The Cancer Hospital Recife. The mean age for this group was 44.8 years (the median: 42 years and the range: 21–81 years). The study group in London-England consisted of 51 women attending either the colposcopy or gynaecological clinics at the Whittington Hospital London. The mean age for this group was 34.7 years (median: 30.5 years and the range: 19–61). In both groups, ethical permission was obtained from the respective hospital's ethics committee after review of the study protocol. All the women in both groups provided informed consent to obtain and use their cervical material for the purpose of the study. Most of the women acknowledged the desire to participate for a long-term follow-up programme. 

This study focuses on women with High-grade Cervical Glandular Intraepithelial Neoplasia (HG CGIN) (adenocarcinoma (AC) precancer lesion, FIGO stages; adenocarcinoma in situ stages 1a1 and 1a2), women with High-grade Cervical Intraepithelial Neoplasia (HG CIN) (squamous cell carcinoma (SCC) precancer lesion; CIN2 and CIN3), and as our control group, women with cervicitis, which is an inflammation of the uterine cervix usually attributed to infection caused by sexually transmitted diseases [18]. Conversely, squamous cell carcinoma is a characteristic malignancy that is thought to originate from the cells that cover the ectocervix, whilst cervical adenocarcinoma is thought to originate from malignant adenomatous or glandular cells distributed along the endocervical canal of the cervix [5–8]. Moreover, cervical adenocarcinoma is rare and accounted for less than 20% of cervical cancer in women as compared to squamous cell carcinoma which accounts for more than 80% of this ailment [5–8]. In this study, we also evaluate the levels of Brn-3a and HPV E6 mRNA in sarcomatoid squamous cell carcinoma, given that it is a very rare, aggressive, and less understood form of cervical cancer [9]. Nonetheless, it is thought to be characterised by biphasic tumour components of squamous cell carcinoma and spindle cell malignancy [10]. Like the other forms of cervical carcinomas in this study, it has also been associated with HPV [5–10]. 

### 2.2. Study Protocol

Cervical cells were obtained and processed using our previously described routine methodology but with some slight modifications to reduce cost, so that it could be conveniently adapted in developing countries [18]. In brief, cervical scrapes were obtained using a spatula and/or cytobrush, and the specimen was placed in 10 mL 1 X PBS and stored on ice for less than 30 minutes prior to a 2-minute 1500 g centrifugation (in developing countries PBS is a cheaper but effective alternative to RPMI media). The concentrated cervical cells recovered were used for RNA extractions. The Brn-3a mRNA and HPV-16 E6 mRNA levels were determined subsequently using our previously described RT-PCR assay [31, 32]. To express the levels of Brn-3a and E6 mRNA, we have taken an arbitrary scale of values in the range of 0.0 to 1.0, with 0.0 representing a lack of detection of mRNA and 1.0 the highest achievable level. 

Importantly, the specimens used in this Material section were not preselected according to their pathological classifications. Indeed, the specimens were obtained by the Clinicians and processed blindly by the Researchers.

## 3. Statistical Analysis

We used the linear regression model (Microsoft Excel) to derive the coefficient of regression between Brn-3a and E6, and the correlation coefficient between Brn-3a and E6 mRNA levels was derived using the Pearson Correlation coefficient (“PEARSON” in Microsoft Excel). The differences of the mean between variables were analysed using Microsoft Excel Student's *t*-test. All other statistical analyses were performed using Microsoft Excel. At all instances, *P* values of less than  .05 (*P* < .05) with 95% Confidence Interval (CI) was used as the cut-off for statistical significance.

## 4. Results and Discussion

**Figure 1:**
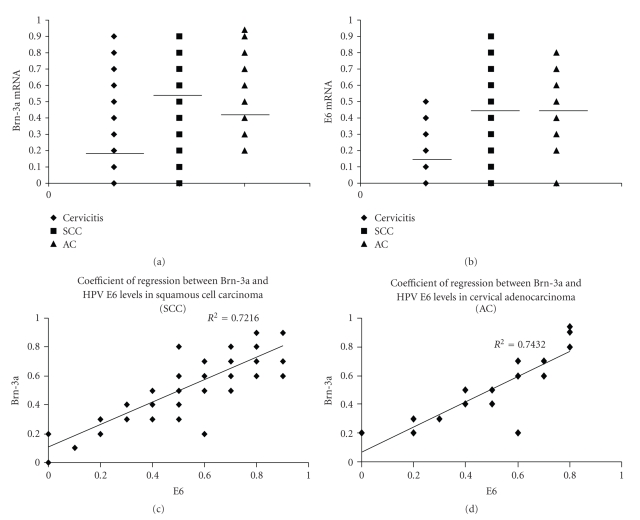
The scatter plot ((a) and (b)) and regression line ((c) and (d)) of Brn-3a and E6 mRNA levels in cervical scrapes obtained from women with High-grade Cervical Intraepithelial Neoplasia (HG CIN) (squamous cell carcinoma (SCC) precancer) and High-grade Cervical Glandular Intraepithelial Neoplasia (HG CGIN) (adenocarcinoma (AC) precancer). Note for the scatter plots, the bar designates the mean Brn-3a and E6 mRNA levels for SCC precancer, AC precancer and cervicitis (as a control group).

The levels of the mRNAs encoding the cellular transcription factor Brn-3a and the transcribed HPV EORF E6 gene product, measured from cervical scrapes of women in Brazil and in England, who had a form of cervical cancer or cervicitis (as a control group), are presented in Table 1. Of the total of 136 cervical smears analysed from both countries, 72, (53%) that is about one in every two, had a form of cervical cancer. Of these, 98.6% (71/72) had significant levels of Brn-3a and 75% (54/72) also had significant E6 (absolute *R* = 0.6), (Figure 1). 

Moreover, 68% (49/72) of patients have SCC precancer of which 48/49 (97.9%) registered high Brn-3a (*P* = .000052) and 33/49 (67.3%) high E6 (*P* = .00046) (Tables 1 and 2). Furthermore, 29% (21/72) had AC precancer with all of them having high Brn-3a 21/21(100%) (*P* = .000026) and (19/21) of them high E6 (*P* = .0001) (Tables 1 and 2). Two patients in Brazil with the very rare sarcomatoid squamous cell carcinoma showed low but detectable levels of Brn-3a and E6, although the low level may represent a problem with the quality of these samples. 

As expected, given that AC and SSCC constitute rare forms of cervical cancer, 33.3% (17/51), or 1 in 3, of the specimens in Brazil were AC precancer, but less than 20% (4/21) of the specimens in England were AC precancer (Table 1). Interestingly though, in the AC precancer specimens in Brazil, Brn-3a was detected in 100% (17/17) of cases, while E6 was detected in 88.2% (15/17) (Table 2). Furthermore, in the Brazilian specimens, 32/51 (62.7%) were SCC precancer, with Brn-3a detected in all of them 100% (32/32) and E6 in 30/32 (96.7%). 

Similar prevalence was also observed with the cases in England (Table 2), where SCC precancer registered 94.5% (17/21) of incidence, of which 94.1% (16/17) had Brn-3a and 76.5% (13/17) had E6. Here, the AC precancer registered a 100% Brn-3a and E6 presence. 

Taken together, we have demonstrated that Brn-3a and HPV E6 mRNA were detected in the cervical scrapes of women with different forms of cervical precancer in both developing and developed countries. This is in accordance with many other studies that have suggested HPV DNA as the primary aetiological agent for these ailments worldwide [1–11], but also support the key role of Brn-3a in activating HPV transcription in different forms of cervical carcinomas. 

To show that Brn-3a mRNA and the HPV E6 mRNA correlated with one another, and could be used as a screening, diagnostic, and prognostic maker for these ailments, a Pearson correlation coefficient was performed and an *r* = 0.85 was obtained. Moreover, the coefficient of regression between Brn-3a and E6 for SCC and AC precancers was respectively deduced to be *R* ^2^ = 0.72 and *R* ^2^ = 0.74 (Figure 1). Thus, a clear correlation exists between the levels of the mRNAs encoding Brn-3a and HPV E6 in different types of cervical lesions. 

**Figure 2:**
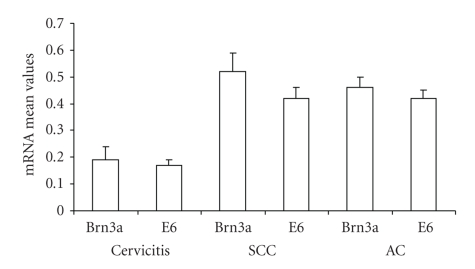
The mean and standard deviation of Brn-3a and E6 mRNA levels in cervical scrapes obtained from women with High-grade Cervical Intraepithelial Neoplasia (HG CIN) (squamous cell carcinoma (SCC) precancer) and High-grade Cervical Glandular Intraepithelial Neoplasia (HG CGIN) (adenocarcinoma (AC) precancer) and cervicitis (as a control group). Note the top bar designates the standard deviation.

In addition, the mean values of Brn-3a and E6 in the cervical scrapes of women with SCC and AC precancers, SSCC and cervicitis as a control group were also determined (Table 1 and Figure 2). The result showed that there is a significant difference between the mean value of Brn-3a for SCC precancer and the control group for women in Recife (*P* = .000001) and for London (*P* = .0003), which is also true for the E6 mean in Recife (*P* = .000002) and for London (*P* = .01). Interestingly, a similar significant difference was observed between the cervicitis control and the AC precancer specimens in Recife (*P* = .00033) for Brn-3a and E6 (*P* = .001). 

In both Brazil and England, the mean value of Brn-3a and E6 in the AC precancer specimens was greater than that of the control group as depicted in Figure 2. This indicates that women with AC and SCC precancers do have high levels of Brn-3a that is directly reflected by the high E6 level (Figure 1, bottom panels), thus, suggesting rapid disease progression as compared to those who though with cervicitis, had no cancer [18]. 

Taken together, this result could be of particular importance for the early diagnosing of rare and common forms of cervical cancers, since the elevated level of Brn-3a occurs uniformly in the cervix, and it is not confined only to the transformation zone, so a high level of Brn-3a in the cervix predicts rapid disease progression upon viral infection. In agreement with this, it could therefore be employed for predicting the early stages of AC in countries where cost is hampering cervical screening programmes, or where classical procedures are inadequate in diagnosing the early stages of SCC and other related cervical diseases. 

This is because, as we have shown in several studies, the rate of transactivation of the oncogenic EORF gene product E6 depends on the level of Brn-3a. Thus, the level of the cellular factor Brn-3a predicts the rate of viral activity, and hence the rate of the accelerated neoplastic transformation. Indeed, Brn-3a measurements have been used successfully in the past in our clinics to recall women for further gynaecological examinations [31, 32]. 

However, further studies are needed to ascertain why young women in a developed country, such as in England, do have high prevalence of SCC precancer (mean age 28.8, Brn-3a mean 0.46 *P* = .001) as compared to women in Brazil a developing country with SCC precancer (mean age 50.7, Brn-3a mean 0.58 *P* = .000001) [18, 31, 32]. In accordance with the literature [6–8, 13–16], the mean age of AC precancer prevalence in Recife, a city in a developing country, was higher (mean age 44, mean Brn-3a 0.47 *P* = .00033) as compared to that observed in London, a city in a developed country (age mean 38.5, mean Brn-3a 0.46) [6–8, 13–16]. 

However, no correlation was observed between age and Brn-3a level in the cervix, suggesting that the high levels of Brn-3a in the cervix of women with cancer in both the developing and developed countries could be due to genetic or other environmental factors rather than hormones [22, 36], which is mostly age related [6–10]. It will be important therefore to carry out a longitudinal study to investigate if women who present high Brn-3a are predisposed to accelerated neoplastic transformation in the cervix should they be infected with HPV, since none of the prophylactic vaccines have a life-time or 100% protection [37]. 

Thus, the expression of the oncogenic HPV cellular transactivator Brn-3a observed in cervical material in this study suggests that it could be used to predict the early onset of cervical adenocarcinoma and squamous cell carcinoma. The few Sarcomatoid squamous cell carcinoma samples available for this study registered the presence of low but detectable levels of Brn-3a and HPV E6 mRNA, and so further work still needs to be done using material obtain from SSCC biopsies, to investigate what role Brn-3a and the transactivated viral oncogenic products play in the natural history of this rare cervical cancer. Thus, studies have shown that, AC, SCC, and even SSCC share a similar aetiological profile that hinges on the expressed oncogenic products of HPV [3, 5–10]. This is in conformity with our findings that, the Brn-3a cellular transcription factor which specifically regulates the production of HPV E6 and E7 oncogenes in cervical cancers could be a pivotal component in the aetiology of these diseases. 

In conclusion, this study suggests that in both in the developed and developing worlds, the cellular factor Brn-3a could be used alongside the oncogenic E6 mRNA in the screening and early diagnosing of both the common and rare types of cervical cancer.

## Figures and Tables

**Table 1:** Patients in Recife-Brazil and London-UK with rare and common forms of cervical precancer lesions plus a cervicitis control group showing the mean mRNA levels of Brn-3a and HPV E6 in cervical scrapes.

Women from Brazil				

Pathology	Case patients.	Age	Brn-3a mRNA	HPV E6 mRNA
	No. (%)	Mean	Mean	Mean

Cervicitis	32 (38.5)	39.4	0.2^a∗^	0.17^⋀^b
SCC precancer	32 (38.5)	50.7	0.58*	0.55^⋀^
			*P = .000001	*P* = .000002
AC precancer	17 (20.5)	44	0.47^a^	0.4^b^
			^a^ *P* = .00033	^b^ *P* = .001
SSCC	2 (2.5)	35	<0.1^†^	<0.1^†^
			^†^0< negligible < 0.1	^†^0< negligible < 0.1

Women from England				

Pathology	Case patients.	Age	Brn-3a mRNA	HPV E6 mRNA
	No (%)	Mean	Mean	Mean

Cervicitis	32 (60.4)	37.2	0.21*	0.17^⋀^
SCC precancer	17 (32.1)	28.8	0.46*	0.30^⋀^
			*P = .0003	^⋀^ *P* = .01
AC precancer	4 (7.5)	38.5	0.46	0.41

SSCC	0	—	—	—

Total women in study				

Pathology	Total case patients.	Age	Brn-3a mRNA	HPV E6 mRNA
	No (%)	Mean	Mean	Mean

Cervicitis	32 + 32 (47.1)	38.3	0.20^∗a^	0.17^⋀^
SCC precancer	32 + 17 (36)	39.8	0.52*	0.42^⋀^
			*P = .000052	^⋀^ *P* = .00046
AC precancer	17 + 4 (15.4)	41.1	0.46^a^	0.42^b^
			^a^ *P* = .000026	^b^ *P* = .0001
SSCC	2 + 0 (1.5)	35	<0.1^†^	<0.1^†^
			^†^0< negligible < 0.1	^†^0< negligible < 0.1

**Table 2:** Presence of Brn-3a and E6 mRNA in the cervix of women in Recife-Brazil with different forms of cervical precancer lesions and a cervicitis control group.

Women from Brazil		

Pathology	Brn-3a mRNA present	HPV E6 mRNA present
(no. of cases)	no. (%)	no. (%)

Cervicitis (32 )	18 (56.3)	15 (46.8)

SCC precancer (32)	32 (100)	30 (93.7)

AC precancer (17)	17 (100)	15 (88.2)

SSCC (2)	2 (100)	2 (100) (%)

Women from England		

Pathology	Brn-3a mRNA present	HPV E6 mRNA present
(no. of cases)	no. (%)	no. (%)

Cervicitis (32)	15 (46.8)	12 (37.5)
SCC precancer (17)	16 (94.1)	13 (76.4)

AC precancer (4)	4 (100)	4 (100)

SSCC (0)	—	—

Total no of pathological cases in Study		

Total pathological cases in	Brn-3a mRNA present	HPV E6 mRNA present
study (no.)	no. (%)	no. (%)

Cervicitis (64)	33 (51.5)	27 (42.2)
SCC precancer (49)	48 (97.9)	33 (67.3)

AC precancer (21)	21 (100)	19 (90.4)

SSCC (2)	2 (100)	2 (100)

## Abbreviations

URR: Upstream Regulatory Region
URR: Upstream Regulatory Region
EORF: Early Open Reading Frame
AC: Cervical Adenocarcinoma
SSCC: Sarcomatoid squamous cell carcinoma of the cervix
SCC: Squamous cell carcinoma of the cervix
HG CGIN: High-grade Cervical Glandular Intraepithelial Neoplasia or AC pre-cancer lesion
HG CIN: High-grade Cervical Intraepithelial Neoplasia or SCC pre-cancer lesion.
